# Mitochondrial Dysfunction and Autophagy in Hepatic Ischemia/Reperfusion Injury

**DOI:** 10.1155/2015/183469

**Published:** 2015-12-06

**Authors:** Kristina L. Go, Sooyeon Lee, Ivan Zendejas, Kevin E. Behrns, Jae-Sung Kim

**Affiliations:** Department of Surgery, University of Florida, Gainesville, FL 32610, USA

## Abstract

Ischemia/reperfusion (I/R) injury remains a major complication of liver resection, transplantation, and hemorrhagic shock. Although the mechanisms that contribute to hepatic I/R are complex and diverse involving the interaction of cell injury in hepatocytes, immune cells, and endothelium, mitochondrial dysfunction is a cardinal event culminating in hepatic reperfusion injury. Mitochondrial autophagy, so-called mitophagy, is a key cellular process that regulates mitochondrial homeostasis and eliminates damaged mitochondria in a timely manner. Growing evidence accumulates that I/R injury is attributed to defective mitophagy. This review aims to summarize the current understanding of autophagy and its role in hepatic I/R injury and highlight the various therapeutic approaches that have been studied to ameliorate injury.

## 1. Introduction

Ischemia/reperfusion (I/R) injury is the phenomenon by which cellular damage in an organ initiated during hypoxia or anoxia becomes exacerbated when oxygen delivery and tissue pH are restored [1]. I/R begins as a localized process leading to an initial parenchymal cell death and progresses to a profound inflammatory response that involves direct and indirect cytotoxic mechanisms [2]. Low-flow states, trauma, liver resection surgery for treatment of benign and malignant disease, and liver transplantation are among the scenarios that predispose the liver to I/R. Liver transplantation is the standard care for patients with the end-stage liver disease and those with irreversible tumors of hepatic origin [3]. However, organ shortage has led to extending the donor selection criteria, including older, steatotic, or non-heart-beating donors as well as organs that have been exposed to extended periods of ischemia [1]. Hepatic I/R remains a source of major complication in clinical practice affecting perioperative morbidity, mortality, and recovery. Despite its profound clinical importance, therapies to suppress I/R at the bedside remain limited largely due to the complex mechanisms that contribute to I/R. In this review, we will discuss the cellular and molecular mechanisms that trigger warm I/R injury and summarize current therapeutic approaches to ameliorate warm I/R injury.

## 2. Types of I/R Injury

The liver is the second largest organ in the body. Due to its highly aerobic nature, as inferred from its unique dual blood supply, liver cells are particularly susceptible to ischemic insult. Hepatic I/R can be categorized into warm and cold ischemia. Whereas warm I/R is observed in vascular occlusion during hepatic resection surgery or during exposure to low-flow incidences such as trauma, hemorrhagic shock, cardiac arrest, and hepatic sinusoidal obstruction syndrome, cold I/R is evident during hepatic transplantation, where the graft is subjected to hypothermic preservation prior to a warm reperfusion phase [4]. Although tissue death is the final outcome from either cold or warm ischemia, the injury mechanisms are quite distinct. For instance, while cold I/R induces injury primarily to sinusoidal endothelium and nonparenchymal cells [5], hepatocytes are a major target of warm I/R injury [6].

Hepatic endothelial and nonparenchymal damage initiates reperfusion injury after cold ischemia. Cellular injury to endothelial and Kupffer cells adversely affects graft microcirculation by increasing platelet activation, vasoconstriction, upregulation of adhesion molecules, and generation of reactive oxygen species (ROS). These events further activate Kupffer cells and recruit neutrophils, ultimately potentiating hepatocyte death [7].

In biochemical aspects, warm ischemia causes three major changes in hepatocytes: (1) anoxia, (2) nutrition depletion, and (3) cytosolic acidosis. The loss of oxygen during ischemia depletes hepatocytes of cellular adenosine triphosphate (ATP), leading to disruption of energy-dependent metabolic and transport processes [6]. Sodium, chloride, and calcium homeostasis, which are tightly regulated by ATP-dependent channels and exchangers, are significantly compromised [6]. Cessation of blood flow likewise results in nutrient depletion and further potentiates ATP loss. Accumulation of lactate and hydrogen ion via anaerobic glycolysis and ATP hydrolysis, respectively, generates the acidic milieu in the cytoplasm, which, in turn, suppresses a myriad of enzymes that optimally operate in a neutral pH [8]. Though prolonged ischemia and severe tissue acidosis eventually cause liver cell death, the acidic environment confers protection to the liver parenchyma during the acute ischemic period [9]. Paradoxically, restoration of blood flow and return of normal pH independently aggravate ischemic damage.

Cold I/R is observed solely in the setting of orthotopic liver transplantation, whereas clinical settings leading to warm I/R are more numerous and occur more frequently. While the reader should be aware that two types of I/R exist and occur through two separate mechanisms, this review will focus on the mechanisms and subsequent therapeutic interventions involved in mitigating warm I/R due to its higher incidence.

## 3. The Mitochondrial Permeability Transition (MPT)

**Figure 1:**
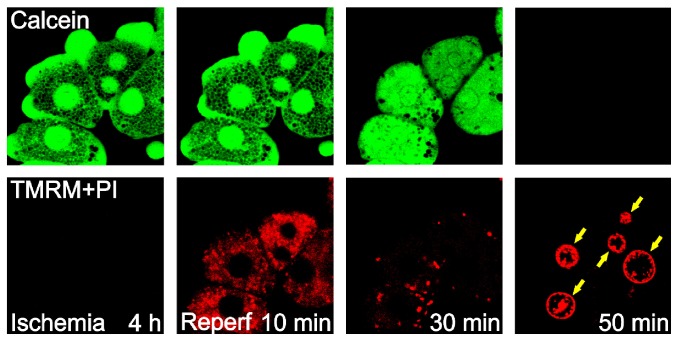
Onset of the MPT after I/R in primary rodent hepatocytes. After 4 hours of simulated ischemia, hepatocytes were reperfused and confocal images of calcein, tetramethylrhodamine methyl ester (TMRM), and propidium iodide (PI, arrows) were simultaneously collected. Polarized mitochondria take up red fluorescing TMRM while simultaneously excluding green fluorescing calcein due to the closed conformation of permeability transition pores. After 4 hours of ischemia, anoxia depolarized the mitochondria and TMRM fluorescence was undetectable. At the same time, the mitochondria in the green channel appeared as dark and round voids where each void represents a single, polarized mitochondrion, indicative of the absence of MPT onset during ischemia. After reperfusion, the mitochondria transiently repolarized within 10 minutes, but the MPT initiated thereafter, as shown by the loss of TMRM fluorescence and diffusion of cytosolic calcein into the mitochondria. Both calcein and TMRM fluorescence completely vanished at 50 minutes and PI labeled the nuclei (arrows) due to the loss of the plasma membrane integrity.

Individual hepatocytes possess hundreds of mitochondria in order to meet the high amount of energy required to execute multiple metabolic functions. While functional mitochondria are absolutely necessary for cell survival and anabolic events, these double-membrane organelles are also causatively involved in both apoptotic and necrotic cell death. When hepatocytes are exposed to oxidative stress, calcium overloading, or I/R, the high conductance permeability transition pores in the mitochondria open and subsequently initiate onset of the MPT [10]. Calcium, inorganic phosphate, alkaline pH, and ROS promote the MPT onset, whereas cyclosporin A (CsA), Mg^2+^, acidic pH, and trifluoperazine prevent the opening of permeability transition pores [11]. Once the MPT initiates, the permeability barrier of the mitochondrial inner membranes collapses and solutes with a molecular mass of up to 1.5 kDa can diffuse freely across the mitochondrial inner membranes. Consequently, mitochondria depolarize, uncouple, and swell, leading to ATP depletion and necrotic cell death (Figure 1). Although necrosis is a predominant type of cell death after I/R, onset of the MPT can also induce apoptosis in ischemic liver and other organs [12]. Rupture of the mitochondrial outer membranes after onset of the MPT and subsequent mitochondrial swelling releases cytochrome *c* that is normally sequestered at the intermembrane space of the mitochondria. Since this 12 kDa protein is an integral element of the apoptosome, the release of cytochrome *c* into the extracellular medium triggers a caspase- and ATP-dependent apoptosis. However, when the cells are depleted of ATP, such as in the setting of ischemia, they do not undergo apoptosis. Instead, these ATP-deficient cells develop necrotic cell death even in the presence of the upstream proapoptotic signals. Thus, ATP availability is a key switch from necrosis to apoptosis [13]. The importance of the MPT in both apoptotic and necrotic hepatocyte death after I/R is further substantiated by the fact that CsA blocks reperfusion-induced apoptosis, whereas tacrolimus, an immunosuppressing agent that does not block the MPT, has no effects on I/R injury [14]. Thus, the MPT is a common pathway leading to both types of cell death after I/R.

Mitochondrial calcium and ROS likely trigger onset of the MPT after I/R. Imaging analysis with isolated rodent hepatocytes shows that increase of mitochondrial calcium and ROS precedes onset of the MPT after reperfusion [15]. Since the chelation of intramitochondrial calcium, but not cytosolic calcium, blocks the generation of ROS and cell death after reperfusion, calcium-mediated mitochondrial ROS formation appears to be the molecular event triggering the MPT after I/R. In contrast, cytosolic ROS play a minimal role in I/R injury, as demonstrated by lack of cytoprotection by apocynin or diphenyleneiodonium chloride, inhibitors of cytosolic NADPH oxidase.

MPT onset remains a critical component in cell death following I/R and therapies to block MPT have been studied. CsA inhibits onset of the MPT by binding to cyclophilin D in the mitochondrial matrix [16]. Although beneficial effects of CsA on reperfusion injury have been documented in animal models [10, 14, 17], its use in human livers remains limited for following reasons. First, CsA has a very narrow therapeutic efficacy of blocking the MPT. High concentrations of CsA cause nephrotoxicity [18]. Second, CsA inhibits calcineurin, provoking immunosuppression. Although nonimmunosuppressive derivatives of CsA such as N-methyl-val-CsA and NIM811 have been introduced [19], their benefits have not been recognized in the clinic. Third, cells can develop unregulated MPT when they are exposed to high concentrations of chemical inducers and excessive stresses [16]. Despite the structural similarity to regulated permeability pores, the unregulated pores do not require calcium for conductance and are not blocked by CsA. It is, however, noteworthy that endeavors are continuously pursued to develop a new class of MPT blockers. For instance, TRO40303 blocks the MPT by binding to the cholesterol site of the peripheral benzodiazepine receptor of the mitochondria [20]. This new agent is currently in clinical trial.

## 4. Autophagy

**Figure 2:**
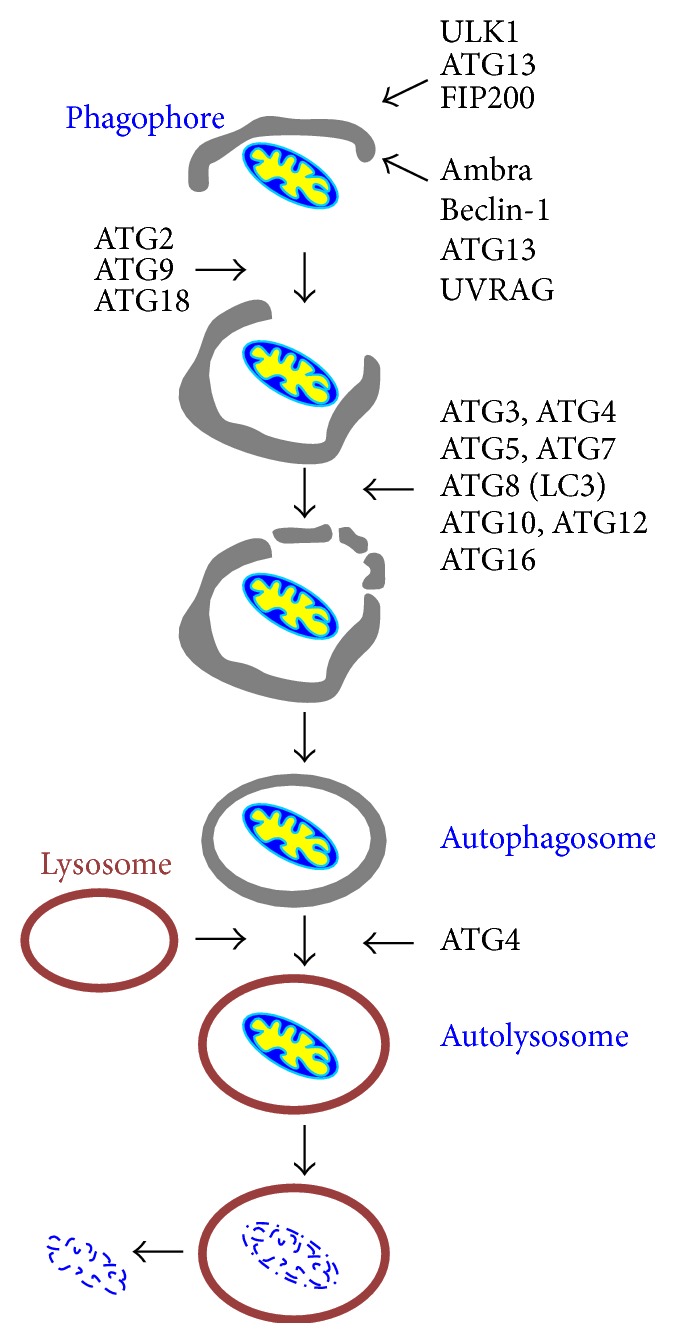
A schematic of autophagy process.

Macroautophagy (referred to as autophagy hereafter) is an intracellular self-digesting pathway to remove abnormal organelles, malformed proteins, and surplus or unnecessary cytoplasmic contents through lysosomal digestion [21]. Autophagy can be divided into five key stages: initiation, nucleation, elongation, fusion, and degradation [22] (Figure 2). Thus far, over thirty autophagy-related (ATG) proteins have been identified in mammalian cells with eighteen designated as core components essential to autophagosome formation [23]. The formation of a double-membrane autophagosome is initiated when core proteins (ULK1, ATG13, FIP200, Ambra, BECN1, ATG13, and UVRAG) act on the phagophore whose membranes are believed to originate from the endoplasmic reticulum, mitochondria, or Golgi [24]. ATG2, ATG3, ATG4, ATG5, ATG7, ATG8, ATG9, ATG10, ATG12, ATG16, and ATG18 guide the formation and subsequent elongation of the double-membrane phagophore. This autophagosome envelops target cellular constituents selectively or nonselectively before fusing with the lysosome to produce a single-membrane autolysosome for degradation. With the help of lysosomal enzymes, autolysosomal contents are degraded into amino acids.

**Figure 3:**
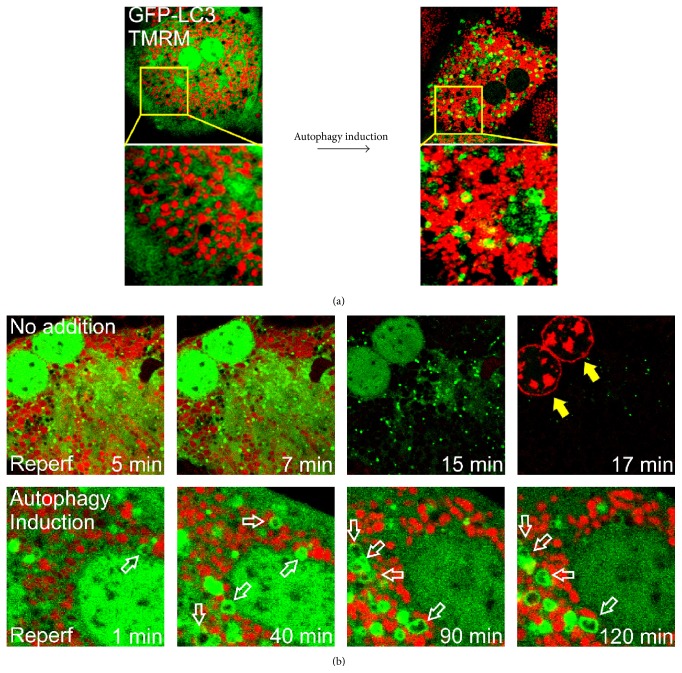
Autophagy in primary mouse hepatocytes. (a) Confocal microscopy with green fluorescent protein-labeled microtubule-associated protein 1 light chain 3 (GFP-LC3) and TMRM in normoxic hepatocytes. Under the basal condition of normoxia, GFP-LC3 predominantly localizes in the cytosol. After autophagy induction, hepatocytes show numerous punctate GFP-LC3, indicative of autophagosomes. Note that some red fluorescing mitochondria are entrapped by GFP-LC3, an event signifying the onset of mitophagy. The bottom panels represent magnified images of the square inserts at the top panels. (b) Loss of autophagy after I/R. Hepatocytes were labeled with GFP-LC3 and subjected to 4 hours of simulated ischemia. After 5 minutes of reperfusion, some autophagosomes (green fluorescing punctate structures) were evident but unable to sequester abnormal mitochondria (top panels). This cell was dead after 17 minutes, as indicated by PI labeling in the nuclei (yellow arrows). In striking contrast, when autophagy was stimulated prior to ischemia, hepatocytes executed a robust autophagy to clear abnormal mitochondria and remained viable after 2 hours of reperfusion. Empty arrows display the autophagosomes surrounding the mitochondria.

Abnormal or dysfunctional mitochondria are cleared through selective autophagy, a process termed mitophagy. In addition to its integral role in the turnover of normal mitochondria, mitophagy prevents accumulation of damaged mitochondria and cytotoxic mitochondrial byproducts [25]. Impaired or insufficient mitophagy can amass damaged mitochondria, leading to uncontrolled increases in ROS, mutations in mitochondrial DNA, energetic failure, and ultimately cell death. There exist at least two distinct types of mitophagy in the cell: phosphatidylinositol-3-kinase- (PI3K-) dependent and phosphatidylinositol-3-kinase- (PI3K-) independent mitophagy. During PI3K-dependent mitophagy, mitochondria sequestered by autophagosomes remain polarized until delivery to the lysosome. Fusion of the autophagosome and lysosome creates the autolysosome [9] (Figure 3(a)). Depolarization occurs later as a result of either the onset of MPT or acidification of the mitophagosomal lumen [15]. This type of autophagy is observed during nutrient starvation and also occurs to hepatocytes after acute ischemic events [9].

PI3K-independent mitophagy becomes evident when the cell experiences a widespread mitochondrial depolarization [9]. This type of mitophagy requires BCL2/adenovirus E1B 19 kDa protein-interacting protein 3 (BNIP3)/BNIP3L (also known as NIX) and PTEN-induced putative kinase protein 1 (PINK1)/Parkin [26]. When the mitochondria are subjected to an uncoupler such as carbonilcyanide p-triflouromethoxyphenylhydrazone (FCCP), PINK1 recruits Parkin to the outer membranes of dysfunctional mitochondria. Recruitment leads to ubiquitination of outer membrane proteins, thereby marking a mitochondrion for mitophagy.

Growing evidence indicates cytoprotective roles of autophagy in various diseases such as aging, diabetes, and neurodegenerative diseases [9]. Autophagy also plays a pivotal role in maintaining mitochondrial function and cell survival after hepatic I/R [8, 25, 27–29]. During ischemia, lack of blood flow exposes the liver to both nutrient depletion and ATP shortage. Although nutrient depletion is one of the most powerful stimuli for autophagy, a near-complete loss of cellular ATP after prolonged ischemia halts the execution of autophagy as this catabolic process relies highly on cellular energy. Moreover, ATP shortage in ischemic livers inhibits ATP-driven calcium pumps and other calcium exchangers, leading to calcium overloading in the cytosol [15]. We have shown that the increase in cytosolic calcium activates calpains which, in turn, hydrolyze key autophagy proteins, ATG7 and BECN1 [27]. Since both proteins are essential for successful formation and elongation of autophagosomes, autophagic machinery in ischemic hepatocytes becomes nonfunctional. Hence, a combination of ATP depletion and ATG loss during prolonged ischemia contributes directly to the reduction of autophagic flux in hepatocytes.

Not only does oxygen supply resume but also hepatocellular pH recovers to normal physiological value after reperfusion. During the early stage of reperfusion, the mitochondria of hepatocytes repolarize transiently (Figure 1). With a reestablishment of the proton motive force within the matrix, the electron transfer chain is reinstituted, albeit temporarily, leading to ATP generation. At this time, autophagy begins to eliminate abnormal proteins and organelles that are produced during ischemia. Consistent with this view, the induction of autophagy is often observed early after an inciting insult [27–31]. In contrast to normoxia or short ischemia where the number of damaged mitochondria is few so that the demand for mitophagy remains minimal, prolonged ischemia and reperfusion lead to substantial calcium and ROS accumulation in a subset of mitochondria. In order for hepatocytes to remain viable, this subset of injured mitochondria must be immediately eliminated via mitophagy. Yet autophagic capacity becomes impaired following prolonged ischemia and subsequent reperfusion due to a detrimental chain of mitochondrial calcium overloading, additional activation of calpains, and loss of autophagy proteins, such that the extent of mitochondrial injury surpasses the ability of autophagy to clear I/R damaged mitochondria. Therefore, at the late stage of reperfusion, autophagy fails to eliminate dysfunctional mitochondria, and widespread onset of the MPT ensues thereafter (Figure 3(b)). The protective role of autophagy in hepatic I/R injury is supported by findings that nutrient depletion prior to ischemia or overexpression of ATG7 and BECN1 all prevents the MPT and promotes hepatocyte survival after reperfusion [27]. It has also been reported that inhibition of autophagy during I/R enhances ROS-induced hepatocyte necrosis [32]. Because defective or insufficient autophagy causatively contributes to lethal I/R injury in the liver, approaches to enhance autophagy or suppress defective autophagy may provide new therapeutic strategies to ameliorate liver function after reperfusion. Indeed, numerous attempts have been made recently to augment autophagy in ischemic livers. For example, studies demonstrate that a low dose of cisplatin, a chemotherapic agent, increases cell viability after warm ischemia through inducing ATG7 and BECN1 expression and more pronounced mitophagy [33, 34]. Pretreatment with rapamycin, an mTOR inhibitor, augments autophagy and reduces hepatic damage in a warm I/R model [35, 36]. Chronic administration of lithium also confers cytoprotection against warm hepatic I/R injury through various mechanisms including enhanced autophagy [37]. Lithium treatment has also been shown to lower inflammatory cytokine production, neutrophil infiltration, and high motility group box 1 (HMGB1) levels in the liver [37]. Carbamazepine, an FDA-approved anticonvulsant drug, provides cytoprotection against I/R in hepatocytes through blockade of a sequential chain of calcium overloading, calpain activation, and depletion of ATG7 and BECN1 [29].

## 5. Nitric Oxide

Nitric oxide (NO), produced from a reaction between L-arginine and oxygen, is a gaseous signaling molecule that plays an important and complex role in hepatic I/R. Two main isoforms, inducible NO synthase (iNOS) and endothelial NOS (eNOS), synthesize NO in the liver during I/R [38]. Whereas eNOS is constitutively expressed on sinusoidal endothelium, iNOS is regulated by many cytokines (TNF-*α* and IL-1) [39]. Depending on cell types and milieu, NO can either promote or prevent cell injury.

Several studies on hepatocytes support a cytoprotective role for NO. In cultured hepatocytes, NO prevents TNF-*α* and Fas ligand-induced apoptosis [40]. During I/R, NO blocks MPT onset and necrotic hepatocellular death through a signaling pathway of guanylyl cyclase and cGMP-dependent kinase (PKG) [41]. Studies demonstrating that NO plays a key role in ischemic preconditioning in cardiomyocytes [42] may also explain how ischemic preconditioning ameliorates I/R in the liver. In addition to its effects on parenchymal cells, NO exerts a protective impact on sinusoids during I/R. Under physiologic conditions, NO induces vasodilation and prevents platelet adhesion, thrombosis, and polymorphonuclear neutrophil accumulation, preventing sinusoidal obstruction [43]. Decreased NO production during ischemia and reperfusion can lead to constriction of the microvascular bed and exacerbate I/R injury. Genetic manipulation of NOS has proposed that inhibition of eNOS exacerbates hepatic I/R [44], whereas eNOS overexpression is cytoprotective [45]. Cytoprotection by eNOS overexpression may be linked to both heme oxygenase-1 (HO-1) and guanylyl cyclase [45]. There are several reports demonstrating that pharmacological increase in NO can reduce hepatic I/R. In cultured hepatocytes, the addition of NO donors such as* S*-nitroso-*N*-acetylpenicillamine (SNAP), DETA NONOate, and spermine NONOate to the reperfusion medium decreases mitochondrial dysfunction and cell necrosis [41]. Mice injected with sildenafil, a cGMP-specific phosphodiesterase inhibitor, or 3-(5′-hydroxymethyl-2′-furyl)-1-benzylindazole (YC-1), a guanylyl cyclase activator, also display lower serum levels of transaminases after I/R [45]. In the clinical setting, patients who have received NO gas during liver transplantation show a faster recovery of liver function and shorter hospital stay with lowered peak serum transaminase levels [46]. Hydrogen sulfide and 17-beta estradiol have been found to increase NO levels in serum and protect against hepatic I/R [47, 48]. The importance of NO is further substantiated by the observation that inhibition of NOS blunts 17-beta estradiol-dependent protection [47]. Beck-Schimmer et al. demonstrate that sevoflurane protects the liver against I/R by increasing mRNA levels of NOS [49].

Although NO can promote cell survival after I/R, cautions should be taken in the use of NO donors. Reactions between superoxide and NO can yield injurious peroxynitrite, a highly reactive oxidant, which can alter DNA and oxidize lipids and proteins [38]. Moreover, accumulation of ROS and inflammatory cytokines during the late stage of reperfusion are known to upregulate iNOS expression, leading to large quantities of NO and subsequent accumulation of reactive nitrogen species. Hence, whether NO promotes or prevents cell death is highly dependent on its concentration, the time and duration of administration, and the condition of the liver including baseline levels of antioxidants available to consume ROS prior to ischemic insult. While the addition of lower concentrations of NO during the early phase of reperfusion may be cytoprotective, high levels, especially at the late stage of reperfusion, may exacerbate tissue injury [50]. Of interest, a recent study suggests that NO impairs autophagy by inhibiting the synthesis of autophagosomes and activating mammalian target of rapamycin (mTOR) [51].

## 6. Surgical Methods to Reduce I/R Injury

### 6.1. Ischemic Preconditioning

During ischemic preconditioning (IPC) of the liver, hepatic inflow is occluded by placing a vascular loop or clamp around the portal triad for an interval of 10–15 minutes followed by removal of the clamp for an additional interval of 10–15 minutes of reperfusion before starting the actual procedure [52]. Various mechanisms have been proposed to explain how IPC provides cytoprotection. Both clinical and basic studies have noted that IPC is associated directly with preservation of ATP after I/R, corroborating the importance of mitochondria in reperfusion injury [53–55]. However, cytoprotective mechanisms underlying IPC are multifactorial, including inhibition of apoptosis and induction of autophagy [55–57]. Liu et al. recently reported that IPC confers protection via enhancing HO-1 mediated autophagy [58]. IPC also causes a mild increase in peroxides which stimulates protective pathways, suggesting a cellular adaptation after exposure to a sublethal oxidative stress [59]. It is noteworthy that inhibition of Kupffer cells with gadolinium chloride abolishes the protective effects of IPC, implying that IPC influences hepatocytes and nonparenchymal cells [60]. Multiple clinical studies have reported that IPC improves outcomes. Using an IPC protocol of 10 minutes of clamping followed by 10 to 15 minutes of reperfusion prior to an anatomic liver resection, Clavien et al. demonstrated that IPC reduced liver injury, indicated by reduced postoperative serum transaminase levels. Furthermore, IPC was associated with less sinusoidal apoptosis in comparison to gender and age matched controls [61]. The benefits of IPC have been substantiated by studies demonstrating that IPC prior to prolonged hepatic inflow occlusion reduced both peak postoperative transaminase levels and the use of intraoperative vasopressors [53, 62]. It is, however, noteworthy that there exist controversies with regard to its efficacy, particularly in surgical trials. While a meta-analysis of the clinical literature has concluded that IPC resulted in reduced hospital length of stay and decreased transfusion rates [63], mortality, morbidity, intraoperative blood loss, and peak transaminase levels were comparable in the two groups. Others have noted that there was no difference in blood loss [64], morbidity, mortality, or lab values [65] in noncirrhotic and cirrhotic livers [66]. The conflicting results seen in the literature may be attributed to differences in IPC protocols used and the heterogeneity of patient populations evaluated, including underlying liver disease prior to resection.

### 6.2. Intermittent Clamping

Like IPC, during intermittent clamping, hepatic inflow is occluded with use of a vascular loop or clamp. Rather than a single period of ischemia prior to one period of reperfusion, a prolonged ischemic period is interrupted by short periods of reperfusion throughout surgery [52]. Belghiti et al. evaluated whether intermittent clamping rather than prolonged, continuous clamping improved surgical outcomes after hepatic resection. In their prospective randomized study, intermittent clamping resulted in reduced peak transaminase levels and was associated with lower frequency of postoperative liver failure [64]. A study comparing IPC and intermittent clamping has concluded that either technique conferred comparable protection based on peak transaminase levels, ICU duration, hospital stay, and complication rate [67]. IPC, however, was associated with lower intraoperative blood loss and shorter transection time. In their murine model, Rüdiger et al. demonstrate that both surgical strategies provide a comparable protection during 75 minutes of hepatic ischemia. Beyond this time, intermittent clamping was superior to IPC [68]. It is, thus, assumed that intermittent clamping confers protection through mechanisms similar to those of IPC, including preservation of mitochondrial integrity and function, and cellular ATP [69].

### 6.3. Remote Ischemic Preconditioning (RIPC)

Remote ischemic preconditioning (RIPC) is the phenomenon whereby brief episodes of I/R to distant tissues or organs such as limb or intestine render the liver resistant to a subsequent sustained I/R. Intestinal I/R prior to hepatic I/R in rats has been reported to improve survival after reperfusion through enhanced expression of HO-1 [70]. RIPC of the femoral bundle also provides protection against liver I/R by upregulating IL-10 and matrix metalloproteinase-8 [71]. Although it is plausible that cytoprotective factors released during this procedure may be delivered to the liver before I/R, future studies are warranted to elucidate the mechanisms and further validate the efficacy of RIPC.

## 7. Diseased Liver and I/R

### 7.1. Role of Aging in I/R

The increase in life expectancy over the past century has resulted in an equivalent rise in elderly patients. Aging is strongly associated with increased incidence and severity of diseases, accidents, and stress. In the liver, aging reduces hepatic blood flow and the number of mitochondria and endoplasmic reticulum (ER). As compared to other organs such as muscles and the brain, deficits in hepatic morphology and function with advancing age are less apparent clinically. The liver is indeed one of the least-studied organs in aging research. Livers from elderly patients, however, have a poorer recovery from surgical stresses during liver resection and transplantation, indicating reduced reparative capacity with aging [72]. The aged liver also responds differently to even minor stresses. For instance, clinical studies evaluating the IPC efficacy have shown that IPC provided greater protection to younger rather than older patients [53, 67]. Several mechanisms may account for this increased susceptibility. Mather and Rottenberg demonstrate that mitochondria isolated from aged livers undergo MPT onset at lower calcium concentrations than their young counterparts, suggesting a reduced calcium buffering capacity in aged mitochondria [73]. Our group has shown the importance of autophagy in the age-dependent mitochondrial injury [28, 74]. While the basal autophagy in aged hepatocytes was comparable to that in young hepatocytes, exposure of aged cells to short ischemia, a condition inducing a minimal injury to young cells, substantially decreased levels of ATG4B which plays an integral role in autophagosome formation and clearance [9]. As a consequence, the autophagy machinery halts, and aged cells fail to remove damaged mitochondria, leading to hepatocellular death later. In addition to autophagy, aging appears to reduce the capacity of proteasomal degradation. Decreased heat shock protein 70 expression and lowered NF-*κ*B activation due to derangements in transport of ubiquitinylated proteins to the proteasome have been observed in senescent liver cells and may account for their enhanced sensitivity to I/R [72, 75, 76].

### 7.2. Role of Steatosis in I/R

Hepatic steatosis, lipid accumulation in the liver that exceeds 5% of wet weight, has become a common problem in industrialized countries caused by multiple comorbidities including obesity, ethanol toxicity, metabolic disorders, and certain drugs [77, 78]. Clinically, steatosis has been associated with increased morbidity and mortality during hepatic resection [79, 80]. The mechanisms underlying the increased susceptibility of steatotic livers to I/R injury may be multifactorial and involve both parenchymal and nonparenchymal dysfunction, including mitochondrial dysfunction, increased lipid peroxidation and ER stress, enhanced increased release of proinflammatory mediators, and increased neutrophil infiltration. In addition, increased cellular volume due to lipid accumulation may potentially obstruct the adjacent sinusoid space leading to poor delivery of oxygen and nutrients and reduction of mitochondrial ATP synthesis. Mitochondrial uncoupling and ATP depletion have been observed in steatotic livers where the expression of mitochondrial uncoupling protein-2 (UCP-2) substantially increases in hepatocytes [81]. As UCP-2 mediates a proton leakage across the mitochondrial inner membranes, steatotic hepatocytes tend to consume oxygen to generate heat rather than ATP. These short-circuited mitochondria encounter more challenges after I/R and become vulnerable to reperfusion injury. Furthermore, steatotic hepatocytes have reduced antioxidant activity, as evidenced by decreased GSH and thioredoxin [82, 83], and are prone to oxidative stress such as I/R injury [84].

While animal literature suggests that fatty livers are more susceptible to I/R injury than lean counterparts, caution should be taken before these studies are translated into clinical application. While genetic and dietary models exist to induce steatosis, no animal model induces both metabolic syndrome and the type of liver pathology seen in human nonalcoholic fatty liver disease (NAFLD) [85, 86].

## 8. The Challenge of Translating Animal Studies to Humans

Table 1 summarizes proposed therapeutic strategies against hepatic reperfusion injury. However, promising treatments in preclinical studies have not translated to significant clinical benefit in human trials. I/R injury is multifactorial. Although simultaneous inhibition of all individual factors triggering I/R injury has intuitive appeal as an ideal therapy, the identification of all these factors is far from complete. Furthermore, it is highly likely that these factors are mechanistically intertwined. There is also a striking disparity between animal models and clinical settings. While animal experiments are typically conducted in young, healthy animals with no comorbidities, the patients requiring liver resection and transplantation are critically ill with preexisting liver diseases. Moreover, the laboratory environment significantly influences experimental results. Thus, despite the importance of animal models in delineating basic principles of therapeutic strategies, they may not encompass all salient features of the human disease condition.

## 9. Concluding Remarks and Future Directions

**Figure 4:**
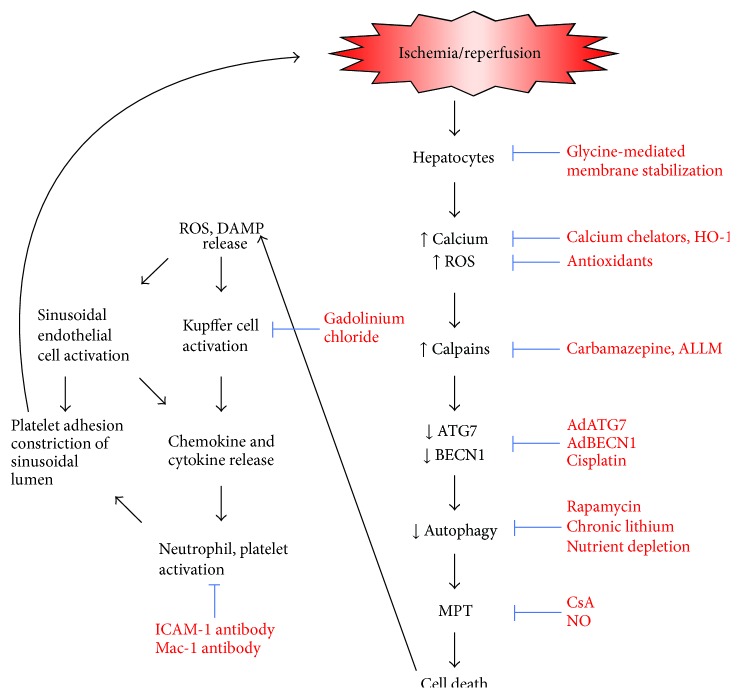
Scheme of I/R-induced impairment of autophagy. After reperfusion, hepatocytes become overloaded with ROS and calcium, which in turn stimulates calpains. These enzymes subsequently hydrolyze ATG7 and BECN1, causing defective autophagy. Since impaired autophagy fails to eliminate abnormal mitochondria, the mitochondria laden with ROS and calcium undergo the MPT and ultimately induce cell death. Suppression of calcium increase, inhibition of calpains with acetyl-leu-leu-methioninal, enhancement of autophagy, or blockade of the MPT with cyclosporin A or nitric oxide prevents reperfusion-induced cell death. Damaged hepatocytes subsequently release damage-associated molecular patterns and ROS. Kupffer cells and sinusoidal endothelial cells are then activated, leading to chemokine and cytokine release, neutrophil and platelet activation, and platelet adhesion to the sinusoidal lumen. Congestion and constriction of the sinusoid further aggravate reperfusion injury. AdATG7, adenovirus expressing ATG7; AdBECN1, adenovirus expressing Beclin-1; ALLM, acetyl-leu-leu-methioninal; CsA, cyclosporin A; DAMP, damage-associated molecular patterns; HO-1, heme oxygenase-1; ICAM-1, intercellular adhesion molecule-1; Mac-1, macrophage-1 antigen; NO, nitric oxide; ROS, reactive oxygen species.

Over the past years, much progress has been made in understanding the mechanisms involved in the development of I/R in the liver. Mitochondrial dysfunction is an important cellular event contributing to I/R injury, and timely removal of abnormal and dysfunction mitochondria not only sustains the quality of the mitochondria but also provides cell and tissue survival (Figure 4). Cells employ mitophagy as an early adaptive response to facilitate a better response to various stresses, including I/R. The mechanisms underlying the onset and propagation of mitophagy remain elusive. Future studies are warranted to characterize detailed signaling pathways inducing lethal reperfusion injury.

## Figures and Tables

**Table 1:** Summary of strategies to reduce I/R injury.

Therapeutic strategy	Proposed mechanism	Reference
Cyclosporin A	Inhibits MPT onset	[87]
ATG7, Beclin-1 overexpression	Increase autophagy	[27]
Carbamazepine	Blocks calpains and increases autophagy	[29]
Hemin	Increases HO-1 Decreases Ca overload and calpain activation Increases autophagy	[88]
Cisplatin	Increases ATG7 and Beclin-1 to increase autophagy Decreases HMGB1 secretion	[33] [34]
Rapamycin	Increases autophagy	[35, 36]
Chronic lithium	Increases autophagy Reduces inflammatory cytokines, neutrophil infiltration, and HMGB1 levels GSK-3*β* inhibitor	[37] [89]
Nutrient depletion	Increases autophagy Reduces circulating HMGB1	[9, 27] [90]
Glutathione	Antioxidant; reduces TNF-*α* levels	[91–93]
*N*-Acetylcysteine	Maintains glutathione	[91, 92]
Glycine	Hepatocyte plasma membrane stabilization	[13, 94]
Gadolinium chloride	Kupffer cell inhibition; reduces lipid peroxidation	[95]
Allopurinol	Xanthine oxidase inhibitor	[96–100]
Interleukin 6	Reduces TNF-*α* levels and attenuates inflammatory response	[101, 102]
Atorvastatin	TLR-4 downregulation and NF-*κ*B downregulation	[103]
Butyrate	Decreases TLR-4 expression	[104]
SB216763	GSK 3*β* inhibitor, suppressing proinflammatory response Inhibits MPT onset	[105]
Oleanolic acid	GSK-3*β* inhibitor	[106]
Ulinastatin	Decreases HMGB1 expression	[107]
Eritoran	TLR-4 antagonist	[108]
ICAM-1 antibody	Blocks neutrophil infiltration	[109]
Mac-1 antibody	Blocks neutrophil activity	[110]
Carbon monoxide	GSK-3*β* inhibitor	[111]
Cobalt protoporphyrin	HO-1 inducer	[112]
Isoflurane	Increases HO-1 activity	[113]
Erythropoietin	Increases HO-1 activity	[114]
Heme oxygenase-1	Increases autophagy Decreases TNF-*α* mediated apoptosis Inhibits TLR4 Maintains sinusoidal diameter and decreases platelet adhesion to sinusoids	[88] [115] [116] [117]
Nitric oxide	Inhibits MPT onset Prevents TNF-*α* and Fas ligand-mediated apoptosis Vasodilator Prevents platelet adhesion	[87] [40] [41] [43]
*S-*Nitroso-*N-*acetylpenicillamine (SNAP)	NO donor	[41]
DETA NONOate	NO donor	[41]
Spermine NONOate	NO donor	[41]
Sildenafil	cGMP phosphodiesterase inhibitor	[45]
YC-1	Guanylyl cyclase activator	[45]
eNOS overexpression	Increases NO	[45]
17-*β* estradiol	Increases serum NO	[47]
Hydrogen sulfide	Increases serum NO	[48]
Sevoflurane	Increases iNOS expression Decreases TNF-*α*, IL-1*β*, MCP-1, NF-*κ*B liver expression, and caspase 3 activity	[49] [118]
Remifentanil	Decreases apoptosis and myeloperoxidase activity Increases superoxide dismutase and NO/iNOS expression	[119]
Ischemic preconditioning	Increases HO-1 Increases autophagy Reduces apoptosis Preserves and restores ATP	[58] [57] [59] [55]
Intermittent clamping	Limits mitochondrial damage Preserves and restores ATP	[69]
Remote ischemic preconditioning	Increases HO-1, IL-10, and MMP-8	[71]

## References

[1] Mendes-Braz M., Elias-Miró M., Jiménez-Castro M. B., Casillas-Ramírez A., Ramalho F. S., Peralta C. (2012). The current state of knowledge of hepatic ischemia-reperfusion injury based on its study in experimental models. *Journal of Biomedicine and Biotechnology*.

[2] Zhai Y., Petrowsky H., Hong J. C., Busuttil R. W., Kupiec-Weglinski J. W. (2013). Ischaemia-reperfusion injury in liver transplantation—from bench to bedside. *Nature Reviews Gastroenterology & Hepatology*.

[3] Wertheim J. A., Petrowsky H., Saab S., Kupiec-Weglinski J. W., Busuttil R. W. (2011). Major challenges limiting liver transplantation in the United States. *American Journal of Transplantation*.

[4] Papadopoulos D., Siempis T., Theodorakou E., Tsoulfas G. (2013). Hepatic ischemia and reperfusion injury and trauma: current concepts. *Archives of Trauma Research*.

[5] Klune J. R., Tsung A. (2010). Molecular biology of liver ischemia/reperfusion injury: established mechanisms and recent advancements. *Surgical Clinics of North America*.

[6] Cursio R., Colosetti P., Gugenheim J. (2015). Autophagy and liver ischemia-reperfusion injury. *BioMed Research International*.

[7] Peralta C., Jiménez-Castro M. B., Gracia-Sancho J. (2013). Hepatic ischemia and reperfusion injury: effects on the liver sinusoidal milieu. *Journal of Hepatology*.

[8] Guan L. Y., Fu P. Y., Li P. D. (2014). Mechanisms of hepatic ischemia-reperfusion injury and protective effects of nitric oxide. *World Journal of Gastrointestinal Surgery*.

[9] Czaja M. J., Ding W.-X., Donohue T. M. (2013). Functions of autophagy in normal and diseased liver. *Autophagy*.

[10] Qian T., Nieminen A.-L., Herman B., Lemasters J. J. (1997). Mitochondrial permeability transition in pH-dependent reperfusion injury to rat hepatocytes. *American Journal of Physiology—Cell Physiology*.

[11] Lemasters J. J., Nieminen A.-L., Qian T. (1998). The mitochondrial permeability transition in cell death: a common mechanism in necrosis, apoptosis and autophagy. *Biochimica et Biophysica Acta*.

[12] Bradham C. A., Qian T., Streetz K., Trautwein C., Brenner D. A., Lemasters J. J. (1998). The mitochondrial permeability transition is required for tumor necrosis factor alpha-mediated apoptosis and cytochrome c release. *Molecular and Cellular Biology*.

[13] Kim J.-S., He L., Qian T., Lemasters J. J. (2003). Role of the mitochondrial permeability transition in apoptotic and necrotic death after ischemia/reperfusion injury to hepatocytes. *Current Molecular Medicine*.

[14] Kantrow S. P., Gierman J. L., Jaligam V. R. (2000). Regulation of tumor necrosis factor cytotoxicity by calcineurin. *FEBS Letters*.

[15] Kim J.-S., Wang J.-H., Lemasters J. J. (2012). Mitochondrial permeability transition in rat hepatocytes after anoxia/reoxygenation: role of Ca^2+^-dependent mitochondrial formation of reactive oxygen species. *The American Journal of Physiology—Gastrointestinal and Liver Physiology*.

[16] He L., Lemasters J. J. (2002). Regulated and unregulated mitochondrial permeability transition pores: a new paradigm of pore structure and function?. *FEBS Letters*.

[17] Hirakawa A., Takeyama N., Nakatani T., Tanaka T. (2003). Mitochondrial permeability transition and cytochrome c release in ischemia-reperfusion injury of the rat liver. *Journal of Surgical Research*.

[18] Klintmalm G. B. G., Iwatsuki S., Starzl T. E. (1981). Nephrotoxicity of cyclosporin A in liver and kidney transplant patients. *The Lancet*.

[19] Waldmeier P. C., Feldtrauer J.-J., Qian T., Lemasters J. J. (2002). Inhibition of the mitochondrial permeability transition by the nonimmunosuppressive cyclosporin derivative NIM811. *Molecular Pharmacology*.

[20] Schaller S., Paradis S., Ngoh G. A. (2010). TRO40303, a new cardioprotective compound, inhibits mitochondrial permeability transition. *Journal of Pharmacology and Experimental Therapeutics*.

[21] Baehrecke E. H. (2005). Autophagy: dual roles in life and death?. *Nature Reviews Molecular Cell Biology*.

[22] Kim K. H., Lee M.-S. (2014). Autophagy—a key player in cellular and body metabolism. *Nature Reviews Endocrinology*.

[23] Vlada C. A., Kim J.-S., Behrns K. E. (2015). Autophagy: self-preservation through cannibalism of proteins and organelles. *Surgery*.

[24] Mijaljica D., Prescott M., Devenish R. J. (2006). Endoplasmic reticulum and golgi complex: contributions to, and turnover by, autophagy. *Traffic*.

[25] Lee S., Kim J.-S. (2014). Mitophagy: therapeutic potentials for liver disease and beyond. *Toxicological Research*.

[26] Gao F., Chen D., Si J. (2015). The mitochondrial protein BNIP3L is the substrate of PARK2 and mediates mitophagy in PINK1/PARK2 pathway. *Human Molecular Genetics*.

[27] Kim J.-S., Nitta T., Mohuczy D. (2008). Impaired autophagy: a mechanism of mitochondrial dysfunction in anoxic rat hepatocytes. *Hepatology*.

[28] Wang J. H., Ahn I. S., Fischer T. D. (2011). Autophagy suppresses age-dependent ischemia and reperfusion injury in livers of mice. *Gastroenterology*.

[29] Kim J.-S., Wang J.-H., Biel T. G. (2013). Carbamazepine suppresses calpain-mediated autophagy impairment after ischemia/reperfusion in mouse livers. *Toxicology and Applied Pharmacology*.

[30] Ding W. X., Li M., Chen X. (2010). Autophagy reduces acute ethanol-induced hepatotoxicity and steatosis in mice. *Gastroenterology*.

[31] Ni H.-M., Bockus A., Boggess N., Jaeschke H., Ding W.-X. (2012). Activation of autophagy protects against acetaminophen-induced hepatotoxicity. *Hepatology*.

[32] Sun K., Xie X., Liu Y. (2013). Autophagy lessens ischemic liver injury by reducing oxidative damage. *Cell and Bioscience*.

[33] Cardinal J., Pan P., Dhupar R. (2009). Cisplatin prevents high mobility group box 1 release and is protective in a murine model of hepatic ischemia/reperfusion injury. *Hepatology*.

[34] Cardinal J., Pan P., Tsung A. (2009). Protective role of cisplatin in ischemic liver injury through induction of autophagy. *Autophagy*.

[35] Evankovich J., Zhang R., Cardinal J. S. (2012). Calcium/calmodulin-dependent protein kinase IV limits organ damage in hepatic ischemia-reperfusion injury through induction of autophagy. *American Journal of Physiology—Gastrointestinal and Liver Physiology*.

[36] Zhu J., Lu T., Yue S. (2015). Rapamycin protection of livers from ischemia and reperfusion injury is dependent on both autophagy induction and mammalian target of rapamycin complex 2-Akt activation. *Transplantation*.

[37] Liu A., Fang H., Dahmen U., Dirsch O. (2013). Chronic lithium treatment protects against liver ischemia/reperfusion injury in rats. *Liver Transplantation*.

[38] Diesen D. L., Kuo P. C. (2010). Nitric oxide and redox regulation in the liver: part I. General considerations and redox biology in hepatitis. *Journal of Surgical Research*.

[39] Geller D. A., Nussler A. K., Di Silvio M. (1993). Cytokines, endotoxin, and glucocorticoids regulate the expression of inducible nitric oxide synthase in hepatocytes. *Proceedings of the National Academy of Sciences of the United States of America*.

[40] Li J., Bombeck C. A., Yang S., Kim Y.-M., Billiar T. R. (1999). Nitric oxide suppresses apoptosis via interrupting caspase activation and mitochondrial dysfunction in cultured hepatocytes. *The Journal of Biological Chemistry*.

[41] Kim J.-S., Ohshima S., Pediaditakis P., Lemasters J. J. (2004). Nitric oxide protects rat hepatocytes against reperfusion injury mediated by the mitochondrial permeability transition. *Hepatology*.

[42] Guo Y., Jones W. K., Xuan Y.-T. (1999). The late phase of ischemic preconditioning is abrogated by targeted disruption of the inducible NO synthase gene. *Proceedings of the National Academy of Sciences of the United States of America*.

[43] Diesen D. L., Kuo P. C. (2011). Nitric oxide and redox regulation in the liver: part II. Redox biology in pathologic hepatocytes and implications for intervention. *Journal of Surgical Research*.

[44] Hines I. N., Kawachi S., Harada H. (2002). Role of nitric oxide in liver ischemia and reperfusion injury. *Molecular and Cellular Biochemistry*.

[45] Duranski M. R., Elrod J. W., Calvert J. W., Bryan N. S., Feelisch M., Lefer D. J. (2006). Genetic overexpression of eNOS attenuates hepatic ischemia-reperfusion injury. *The American Journal of Physiology—Heart and Circulatory Physiology*.

[46] Lang J. D., Teng X., Chumley P. (2007). Inhaled NO accelerates restoration of liver function in adults following orthotopic liver transplantation. *The Journal of Clinical Investigation*.

[47] Eckhoff D. E., Bilbao G., Frenette L., Thompson J. A., Contreras J. L. (2002). 17-Beta-estradiol protects the liver against warm ischemia/reperfusion injury and is associated with increased serum nitric oxide and decreased tumor necrosis factor-alpha. *Surgery*.

[48] King A. L., Polhemus D. J., Bhushan S. (2014). Hydrogen sulfide cytoprotective signaling is endothelial nitric oxide synthase-nitric oxide dependent. *Proceedings of the National Academy of Sciences of the United States of America*.

[49] Beck-Schimmer B., Breitenstein S., Urech S. (2008). A randomized controlled trial on pharmacological preconditioning in liver surgery using a volatile anesthetic. *Annals of Surgery*.

[50] Chung H.-T., Pae H.-O., Choi B.-M., Billiar T. R., Kim Y.-M. (2001). Nitric oxide as a bioregulator of apoptosis. *Biochemical and Biophysical Research Communications*.

[51] Sarkar S., Korolchuk V. I., Renna M. (2011). Complex inhibitory effects of nitric oxide on autophagy. *Molecular Cell*.

[52] Selzner N., Rudiger H., Graf R., Clavien P.-A. (2003). Protective strategies against ischemic injury of the liver. *Gastroenterology*.

[53] Clavien P.-A., Selzner M., Rüdiger H. A. (2003). A prospective randomized study in 100 consecutive patients undergoing major liver resection with versus without ischemic preconditioning. *Annals of Surgery*.

[54] Lee W.-Y., Lee S.-M. (2006). Synergistic protective effect of ischemic preconditioning and allopurinol on ischemia/reperfusion injury in rat liver. *Biochemical and Biophysical Research Communications*.

[55] Selzner N., Selzner M., Jochum W., Clavien P.-A. (2003). Ischemic preconditioning protects the steatotic mouse liver against reperfusion injury: an ATP dependent mechanism. *Journal of Hepatology*.

[56] Ko J. S., Gwak M. S., Kim G. S. (2013). The protective effect of ischemic preconditioning against hepatic ischemic-reperfusion injury under isoflurane anesthesia in rats. *Transplantation Proceedings*.

[57] Yadav S. S., Sindram D., Perry D. K., Clavien P.-A. (1999). Ischemic preconditioning protects the mouse liver by inhibition of apoptosis through a caspase-dependent pathway. *Hepatology*.

[58] Liu A., Fang H., Wei W., Dirsch O., Dahmen U. (2014). Ischemic preconditioning protects against liver ischemia/reperfusion injury via heme oxygenase-1-mediated autophagy. *Critical Care Medicine*.

[59] Rüdiger H. A., Graf R., Clavien P.-A. (2003). Sub-lethal oxidative stress triggers the protective effects of ischemic preconditioning in the mouse liver. *Journal of Hepatology*.

[60] Tejima K., Arai M., Ikeda H. (2004). Ischemic preconditioning protects hepatocytes via reactive oxygen species derived from Kupffer cells in rats. *Gastroenterology*.

[61] Clavien P.-A., Yadav S., Sindram D., Bentley R. C. (2000). Protective effects of ischemic preconditioning for liver resection performed under inflow occlusion in humans. *Annals of Surgery*.

[62] Choukèr A., Schachtner T., Schauer R. (2004). Effects of Pringle manoeuvre and ischaemic preconditioning on haemodynamic stability in patients undergoing elective hepatectomy: a randomized trial. *British Journal of Anaesthesia*.

[63] O'Neill S., Leuschner S., McNally S. J., Garden O. J., Wigmore S. J., Harrison E. M. (2013). Meta-analysis of ischaemic preconditioning for liver resections. *British Journal of Surgery*.

[64] Belghiti J., Noun R., Malafosse R. (1999). Continuous versus intermittent portal triad clamping for liver resection: a controlled study. *Annals of Surgery*.

[65] Ye B., Zhao H., Hou H. (2014). Ischemic preconditioning provides no additive clinical value in liver resection of cirrhotic and non-cirrhotic patients under portal triad clamping: a prospective randomized controlled trial. *Clinics and Research in Hepatology and Gastroenterology*.

[66] Capussotti L., Nuzzo G., Polastri R., Giuliante F., Muratore A., Giovannini I. (2003). Continuous versus intermittent portal triad clamping during hepatectomy in cirrhosis. Results of a prospective, randomized clinical trial. *Hepato-Gastroenterology*.

[67] Petrowsky H., McCormack L., Trujillo M., Selzner M., Jochum W., Clavien P.-A. (2006). A prospective, randomized, controlled trial comparing intermittent portal triad clamping versus ischemic preconditioning with continuous clamping for major liver resection. *Annals of Surgery*.

[68] Rüdiger H. A., Kang K.-J., Sindram D., Riehle H.-M., Clavien P.-A. (2002). Comparison of ischemic preconditioning and intermittent and continuous inflow occlusion in the murine liver. *Annals of Surgery*.

[69] Ben Mosbah I., Duval H., Mbatchi S.-F. (2014). Intermittent selective clamping improves rat liver regeneration by attenuating oxidative and endoplasmic reticulum stress. *Cell Death and Disease*.

[70] Kageyama S., Hata K., Tanaka H. (2015). Intestinal ischemic preconditioning ameliorates hepatic ischemia/reperfusion injury in rats: role of heme oxygenase 1 in the second window of protection. *Liver Transplantation*.

[71] Oberkofler C. E., Limani P., Jang J.-H. (2014). Systemic protection through remote ischemic preconditioning is spread by platelet-dependent signaling in mice. *Hepatology*.

[72] Okaya T., Blanchard J., Schuster R. (2005). Age-dependent responses to hepatic ischemia/reperfusion injury. *Shock*.

[73] Mather M., Rottenberg H. (2000). Aging enhances the activation of the permeability transition pore in mitochondria. *Biochemical and Biophysical Research Communications*.

[74] Wang J.-H., Behrns K. E., Leeuwenburgh C., Kim J.-S. (2012). Critical role of autophagy in ischemia/reperfusion injury to aged livers. *Autophagy*.

[75] Huber N., Sakai N., Eismann T. (2009). Age-related decrease in proteasome expression contributes to defective nuclear factor-*κ*B activation during hepatic ischemia/reperfusion. *Hepatology*.

[76] Wang X. H., Wang K., Zhang F. (2004). Alleviating ischemia-reperfusion injury in aged rat liver by induction of heme oxygenase-1. *Transplantation Proceedings*.

[77] Hornbøll P., Olsen T. S. (1982). Fatty changes in the liver: the relation to age, overweight and diabetes mellitus. *Acta Pathologica, Microbiologica, et Immunologica Scandinavica Section A: Pathology*.

[78] Berson A., De Beco V., Lettéron P. (1998). Steatohepatitis-inducing drugs cause mitochondrial dysfunction and lipid peroxidation in rat hepatocytes. *Gastroenterology*.

[79] Behrns K. E., Tsiotos G. G., DeSouza N. F., Krishna M. K., Ludwig J., Nagorney D. M. (1998). Hepatic steatosis as a potential risk factor for major hepatic resection. *Journal of Gastrointestinal Surgery*.

[80] Veteläinen R., van Vliet A., Gouma D. J., van Gulik T. M. (2007). Steatosis as a risk factor in liver surgery. *Annals of Surgery*.

[81] Evans Z. P., Ellett J. D., Schmidt M. G., Schnellmann R. G., Chavin K. D. (2008). Mitochondrial uncoupling protein-2 mediates steatotic liver injury following ischemia/reperfusion. *Journal of Biological Chemistry*.

[82] Tashiro H., Kuroda S., Mikuriya Y., Ohdan H. (2014). Ischemia-reperfusion injury in patients with fatty liver and the clinical impact of steatotic liver on hepatic surgery. *Surgery Today*.

[83] Zhang C., Huang C., Tian Y., Li X. (2014). Polyol pathway exacerbated ischemia/reperfusion-induced injury in steatotic liver. *Oxidative Medicine and Cellular Longevity*.

[84] Selzner M., RüDiger H. A., Sindram D., Madden J., Clavien P.-A. (2000). Mechanisms of ischemic injury are different in the steatotic and normal rat liver. *Hepatology*.

[85] Chu M. J. J., Hickey A. J. R., Phillips A. R. J., Bartlett A. S. J. R. (2013). The impact of hepatic steatosis on hepatic ischemia-reperfusion injury in experimental studies: a systematic review. *BioMed Research International*.

[86] Selzner N., Selzner M., Jochum W., Amann-Vesti B., Graf R., Clavien P.-A. (2006). Mouse livers with macrosteatosis are more susceptible to normothermic ischemic injury than those with microsteatosis. *Journal of Hepatology*.

[87] Kim J.-S., Ohshima S., Pediaditakis P., Lemasters J. J. (2004). Nitric oxide: a signaling molecule against mitochondrial permeability transition- and pH-dependent cell death after reperfusion. *Free Radical Biology and Medicine*.

[88] Yun N., Cho H.-I., Lee S.-M. (2014). Impaired autophagy contributes to hepatocellular damage during ischemia/reperfusion: heme oxygenase-1 as a possible regulator. *Free Radical Biology and Medicine*.

[89] Xia Y., Rao J., Yao A. (2012). Lithium exacerbates hepatic ischemia/reperfusion injury by inhibiting GSK-3*β*/NF-*κ*B-mediated protective signaling in mice. *European Journal of Pharmacology*.

[90] Rickenbacher A., Jang J. H., Limani P. (2014). Fasting protects liver from ischemic injury through Sirt1-mediated downregulation of circulating HMGB1 in mice. *Journal of Hepatology*.

[91] Nakano H., Boudjema K., Alexandre E. (1995). Protective effects of N-acetylcysteine on hypothermic ischemia-reperfusion injury of rat liver. *Hepatology*.

[92] Fukuzawa K., Emre S., Senyuz O., Acarli K., Schwartz M. E., Miller C. M. (1995). N-acetylcysteine ameliorates reperfusion injury after warm hepatic ischemia. *Transplantation*.

[93] Suyavaran A., Ramamurthy C., Mareeswaran R., Subastri A., Lokeswara Rao P., Thirunavukkarasu C. (2015). TNF-*α* suppression by glutathione preconditioning attenuates hepatic ischemia reperfusion injury in young and aged rats. *Inflammation Research*.

[94] Nishimura Y., Romer L. H., Lemasters J. J. (1998). Mitochondrial dysfunction and cytoskeletal disruption during chemical hypoxia to cultured rat hepatic sinusoidal endothelial cells: the pH paradox and cytoprotection by glucose, acidotic pH, and glycine. *Hepatology*.

[95] Giakoustidis D. E., Iliadis S., Tsantilas D. (2003). Blockade of Kupffer cells by gadolinium chloride reduces lipid peroxidation and protects liver from ischemia/reperfusion injury. *Hepato-Gastroenterology*.

[96] Wiezorek J. S., Brown D. H., Kupperman D. E., Brass C. A. (1994). Rapid conversion to high xanthine oxidase activity in viable Kupffer cells during hypoxia. *Journal of Clinical Investigation*.

[97] Jeon B.-R., Yeom D.-H., Lee S.-M. (2001). Protective effect of allopurinol on hepatic energy metabolism in ischemic and reperfused rat liver. *Shock*.

[98] Taha M. O., Simões M. J., Noguerol E. C. (2009). Effects of allopurinol on ischemia and reperfusion in rabbit livers. *Transplantation Proceedings*.

[99] Peglow S., Toledo A. H., Anaya-Prado R., Lopez-Neblina F., Toledo-Pereyra L. H. (2011). Allopurinol and xanthine oxidase inhibition in liver ischemia reperfusion. *Journal of Hepato-Biliary-Pancreatic Sciences*.

[100] Liu P.-G., He S.-Q., Zhang Y.-H., Wu J. (2008). Protective effects of apocynin and allopurinol on ischemia/reperfusion-induced liver injury in mice. *World Journal of Gastroenterology*.

[101] Camargo C. A., Madden J. F., Gao W., Selvan R. S., Clavien P.-A. (1997). Interleukin-6 protects liver against warm ischemia/reperfusion injury and promotes hepatocyte proliferation in the rodent. *Hepatology*.

[102] Selzner N., Selzner M., Tian Y., Kadry Z., Clavien P.-A. (2002). Cold ischemia decreases liver regeneration after partial liver transplantation in the rat: a TNF-*α*/IL-6-dependent mechanism. *Hepatology*.

[103] Ajamieh H., Farrell G., Wong H. J. (2012). Atorvastatin protects obese mice against hepatic ischemia-reperfusion injury by Toll-like receptor-4 suppression and endothelial nitric oxide synthase activation. *Journal of Gastroenterology and Hepatology*.

[104] Liu B., Qian J., Wang Q., Wang F., Ma Z., Qiao Y. (2014). Butyrate protects rat liver against total hepatic ischemia reperfusion injury with bowel congestion. *PLoS ONE*.

[105] Ren F., Duan Z., Cheng Q. (2011). Inhibition of glycogen synthase kinase 3 beta ameliorates liver ischemia reperfusion injury by way of an interleukin-10-mediated immune regulatory mechanism. *Hepatology*.

[106] Gui B., Hua F., Chen J., Xu Z., Sun H., Qian Y. (2014). Protective effects of pretreatment with oleanolic acid in rats in the acute phase of hepatic ischemia-reperfusion injury: role of the PI3K/Akt pathway. *Mediators of Inflammation*.

[107] Tong Y., Tang Z., Yang T. (2014). Ulinastatin preconditioning attenuates inflammatory reaction of hepatic ischemia reperfusion injury in rats via high mobility group box 1(HMGB1) inhibition. *International Journal of Medical Sciences*.

[108] Mcdonald K. A., Huang H., Tohme S. (2015). Toll-like receptor 4 (TLR4) antagonist eritoran tetrasodium attenuates liver ischemia and reperfusion injury through inhibition of high-mobility group box protein B1 (HMGB1). *Molecular Medicine*.

[109] Nakano H., Nagasaki H., Barama A. (1997). The effects of N-acetylcysteine and anti-intercellular adhesion molecule-1 monoclonal antibody against ischemia-reperfusion injury of the rat steatotic liver produced by a choline-methionine-deficient diet. *Hepatology*.

[110] Jaeschke H., Farhood A., Bautista A. P., Spolarics Z., Spitzer J. J., Smith C. W. (1993). Functional inactivation of neutrophils with a Mac-1 (CD11b/CD18) monoclonal antibody protects against ischemia-reperfusion injury in rat liver. *Hepatology*.

[111] Kim H. J., Joe Y., Kong J. S. (2013). Carbon monoxide protects against hepatic ischemia/reperfusion injury via ROS-dependent akt signaling and inhibition of glycogen synthase kinase 3*β*. *Oxidative Medicine and Cellular Longevity*.

[112] Tamura T., Kondo T., Ogawa K., Fukunaga K., Ohkohchi N. (2013). Protective effect of heme oxygenase-1 on hepatic ischemia-reperfusion injury through inhibition of platelet adhesion to the sinusoids. *Journal of Gastroenterology and Hepatology*.

[113] Lv X., Yang L., Tao K. (2011). Isoflurane preconditioning at clinically relevant doses induce protective effects of heme oxygenase-1 on hepatic ischemia reperfusion in rats. *BMC Gastroenterology*.

[114] Riehle K. J., Hoagland V., Benz W., Campbell J. S., Liggitt D. H., Langdale L. A. (2014). Hepatocellular heme oxygenase-1: a potential mechanism of erythropoietin-mediated protection after liver ischemia-reperfusion injury. *Shock*.

[115] Kim S.-J., Eum H.-A., Billiar T. R., Lee S.-M. (2013). Role of heme oxygenase 1 in TNF/TNF receptor-mediated apoptosis after hepatic ischemia/reperfusion in rats. *Shock*.

[116] Huang H. F., Zeng Z., Wang K. H. (2015). Heme oxygenase-1 protects rat liver against warm ischemia/reperfusion injury via TLR2/TLR4-triggered signaling pathways. *World Journal of Gastroenterology*.

[117] Wunder C., Scott J. R., Lush C. W. (2004). Heme oxygenase modulates hepatic leukocyte sequestration via changes in sinusoidal tone in systemic inflammation in mice. *Microvascular Research*.

[118] Rancan L., Huerta L., Cusati G. (2014). Sevoflurane prevents liver inflammatory response induced by lung ischemia-reperfusion. *Transplantation*.

[119] Yang L.-Q., Tao K.-M., Liu Y.-T. (2011). Remifentanil preconditioning reduces hepatic ischemia-reperfusion injury in rats via inducible nitric oxide synthase expression. *Anesthesiology*.

